# 2,3,7,8-Tetrachlorodibenzo-*p*-dioxin modifies alternative splicing in mouse liver

**DOI:** 10.1371/journal.pone.0219747

**Published:** 2019-08-06

**Authors:** Ana B. Villaseñor-Altamirano, John D. Watson, Stephenie D. Prokopec, Cindy Q. Yao, Paul C. Boutros, Raimo Pohjanvirta, Jesús Valdés-Flores, Guillermo Elizondo

**Affiliations:** 1 Cell Biology Department, Center for Research and Advanced Studies of the National Polytechnic Institute, CINVESTAN-IPN, Mexico City, Mexico; 2 International Laboratory for Human Genome Research, National Autonomous University of Mexico, Queretaro, Mexico; 3 Ontario Institute for Cancer Research, Toronto, Canada; 4 Department of Medical Biophysics, University of Toronto, Toronto, Canada; 5 Department of Human Genetics, University of California, Los Angeles, Los Angeles, California, United States of America; 6 Laboratory of Toxicology, National Institute for Health and Welfare, Kuopio, Finland; 7 Department of Food Hygiene and Environmental Health, University of Helsinki, Helsinki, Finland; 8 Biochemistry Department, Center for Research and Advanced Studies of the National Polytechnic Institute, CINVESTAV-IPN, Mexico City, Mexico; University of Nebraska-Lincoln, UNITED STATES

## Abstract

Alternative splicing is a co-transcriptional mechanism that generates protein diversity by including or excluding exons in different combinations, thereby expanding the diversity of protein isoforms of a single gene. Abnormalities in this process can result in deleterious effects to human health, and several xenobiotics are known to interfere with splicing regulation through multiple mechanisms. These changes could lead to human diseases such as cancer, neurological disorders, autoimmune diseases, and developmental disorders. 2,3,7,8-Tetrachlorodibenzo-*p*-dioxin (TCDD) is an environmental contaminant generated as a byproduct of various industrial activities. Exposure to this dioxin has been linked to a wide range of pathologies through the alteration of multiple cellular processes. However, the effects of TCDD exposure on alternative splicing have not yet been studied. Here, we investigated whether a single po. dose of 5 μg/kg or 500 μg/kg TCDD influence hepatic alternative splicing in adult male C57BL/6Kou mouse. We identified several genes whose alternative splicing of precursor messenger RNAs was modified following TCDD exposure. In particular, we demonstrated that alternative splicing of *Cyp1a1*, *Ahrr*, and *Actn1* was significantly altered after TCDD treatment. These findings show that the exposure to TCDD has an impact on alternative-splicing, and suggest a new avenue for understanding TCDD-mediated toxicity and pathogenesis.

## Introduction

Dioxins are a group of environmentally persistent contaminants, which include polychlorinated dibenzo-*p*-dioxins (PCDDs), polychlorinated dibenzofurans (PCDFs) and 11 dioxin-like polychlorinated biphenyls (PCBs). 2,3,7,8-Tetrachlorodibenzo-*p*-dioxin (TCDD) is the most toxic dioxin and is a product of anthropogenic activity generated from waste incineration, chlorine paper bleaching and pesticide manufacture [1]. Human exposure to TCDD has been linked to a variety of malignancies, such as chloracne and hepatotoxicity [2]. In animal models, TCDD exposure produces an exceedingly broad range of toxic effects including wasting syndrome, thymic atrophy, endocrine disruption, immune suppression, behavioral alterations, teratogenicity, cancer and death [3, 4]. The precise mechanisms through which TCDD induces toxicity are not fully understood; however, almost all of its effects are mediated through the aryl hydrocarbon receptor (AhR), a ligand-dependent transcription factor that is a member of the bHLH-PAS (basic-helix-loop-helix-Per-ARNT-Sim) superfamily. Upon binding to TCDD, AhR activates two pathways. The genomic pathway as a transcription factor, binding to xenobiotic response elements (XREs) located in the promoter regions of its target genes, resulting in an up- or down-regulation of the abundance of a battery of genes encoding xenobiotic-metabolizing enzymes [5] among many others [6–8]. And the nongenomic pathway, exerted through a variety of ways, including crosstalk with other transcription factor (such as estrogen receptor alpha, androgen receptor, NF-κB, and ß-catenin) [9–12], altering histone marks or DNA methylation patterns [13, 14], modifying the activation of Src tyrosine kinase [15], or increasing the intracellular calcium concentration [16].

Benzo[*a*]pyrene, an AhR agonist, disrupt nephrogenesis by modulating the splicing of Wilms’s tumor suppressor [17]. Moreover, CD44 splicing variants were found after Benzo[*a*]pyrene treatment [18]. On the other hand, the effects of TCDD exposure on alternative splicing mechanisms have not been investigated, nor more broadly has the influence of AhR activation. However, there are some observations that suggest it. Human *ALDH3* gene present three alternative splice acceptor sites at the 3’-end of intron 1. Interestingly, the splice variants derived from these sites were observed in controls but not in HepG2-TCDD treated cells, suggesting that this dioxin might modify alternative splicing [19]. Alternative splicing occurs co-transcriptionally and increases protein diversity by including or excluding exons in different combinations, resulting in the production of a diverse array of proteins from a single gene. In humans, ~92–94% of genes are alternatively spliced, allowing for the generation of more than 100,000 protein isoforms [20, 21]. This process is carried out by the splicing code, which consists of three elements: the *cis*-acting elements of the transcript, the spliceosome (which assembles step-wise on the precursor mRNA (pre-mRNA) substrates), and auxiliary factors. Each of these components can be targeted by external agents, causing modifications to the alternative splicing of pre-mRNAs. In particular, xenobiotics can interfere with splicing through multiple mechanisms including inhibition of spliceosome assembly, interference with the recognition of alternative exons, or modification of the abundances of splicing factors [22]. Alterations in the splicing process can result in significant deleterious effects on human health. In particular, alternative splicing abnormalities have been linked to cancer, neurological disorders, autoimmune diseases, and developmental disorders, among others [23, 24]. Therefore, identifying agents with the potential to alter alternative splicing is crucial for understanding many human health problems.

To address this, we have investigated the effects of TCDD exposure on alternative splicing using a mouse model known to be sensitive to TCDD toxicities. In particular, we have evaluated the transcriptome of liver from adult male mice exposed to a low or high dose of TCDD (5 or 500 μg/kg) or vehicle control, focusing on differential abundance of alternatively spliced transcripts. We identified several genes whose splicing processes were modified by TCDD exposure and performed validation of key findings using real-time quantitative PCR (qPCR).

## Materials and methods

### Animal handling

Male C57BL/6Kou mice at the age of 13–15 weeks were obtained from the National Public Health Institute, Division of Environmental Health Institute, Kuopio, Finland. The animals were housed in a pathogen-free facility at 21°C with 50 ± 10% relative humidity and artificial illumination on a 12/12 hour light/dark cycle and tap water *ad libitum*. This study was approved by the Finnish National Animal Experiment Board (Eläinkoelautakunta, ELLA; permit code: ESLH-2008-07223/Ym-23).

### Experimental design

**Fig 1:**
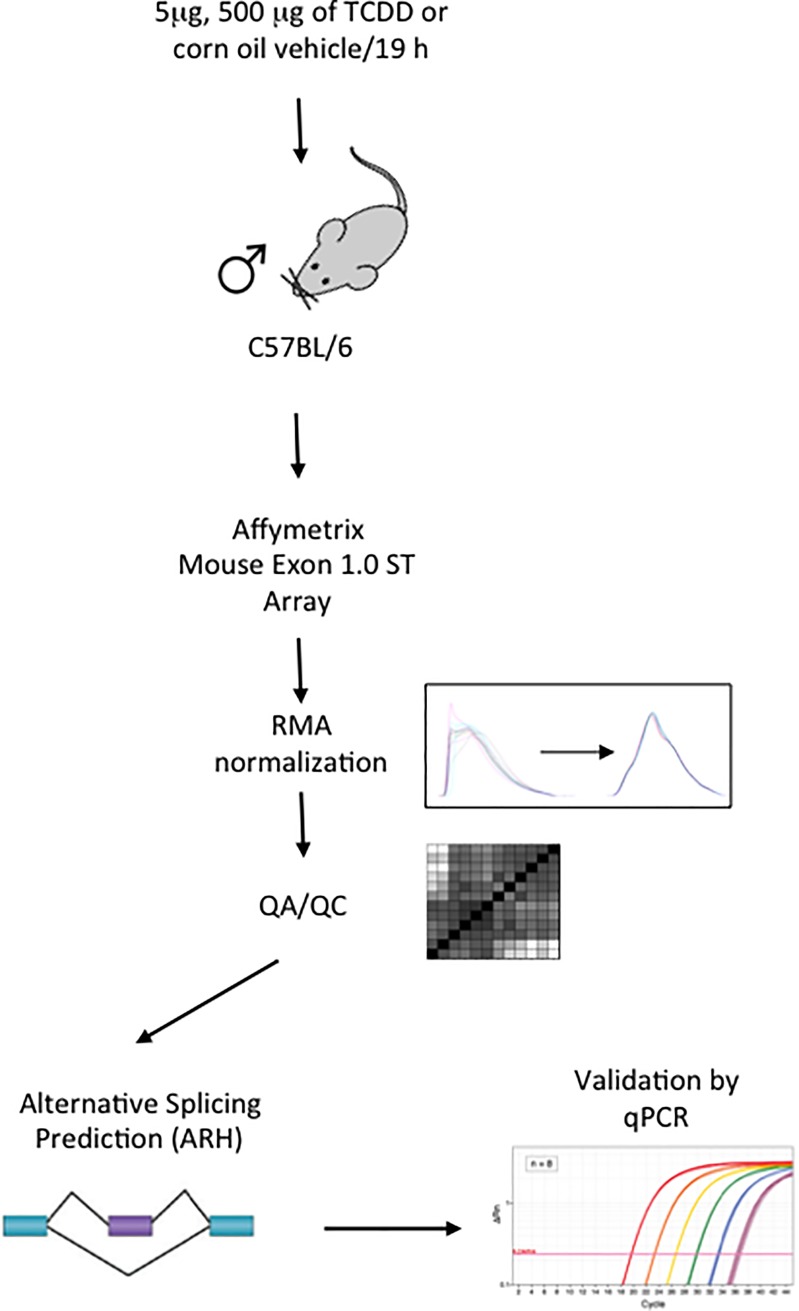
Experimental design. Twelve total male C57BL/6Kou mice at the age of 13–15 weeks were divided into 3 groups of 4 mice each, and treated by oral gavage with a 5 or 500 μg/kg dose of TCDD dissolved in corn oil or corn oil vehicle alone (10 mL/kg). Liver tissue was collected 19 h post-treatment. Affymetrix Mouse Exon 1.0 ST Arrays were used to assess RNA abundance. Data were normalized using RMA and homogeneity assessed; no outliers were detected. Alternative splicing events were predicted using an entropy base measure (ARH), and transcripts with significantly differential abundance were validated using RT-qPCR.

Animals were divided into 3 groups of 4 mice each and treated by oral gavage with a 5 μg/kg or 500 μg/kg dose of TCDD dissolved in corn oil or corn oil alone (10 mL/kg). Mice were observed 2 times after exposure for their general appearance and behavior. Animals were euthanized at 19 hours post-treatment with carbon dioxide followed immediately by cardiac exsanguination. Livers were immediately excised and frozen in liquid nitrogen. The overall experimental design is outlined in Fig 1.

### RNA isolation and exon array hybridization

Liver samples were ground to a fine powder in liquid nitrogen using a mortar and pestle, followed by homogenization in a lysis buffer using a Polytron. RNA was isolated using an RNeasy Mini Kit (Qiagen, Mississauga, Canada) following the manufacturer’s instructions. RNA was quantified using a NanoDrop UV spectrophotometer (Thermo Scientific, Mississauga, Canada) and its integrity verified using RNA 6000 Nano chips on an Agilent 2100 Bioanalyzer (Agilent Technologies, Mississauga, Canada). All samples with an RNA integrity number (RNI) ≥ 8.5 were used for subsequent analysis. RNA was assayed on an Affymetrix Mouse Exon 1.0 ST Array at the Center for the Applied Genomics at the Hospital for Sick Children (Toronto, Canada) according to manufacturer’s protocols.

### Exon array preprocessing

Raw array data (CEL files) were loaded into the R statistical environment (v3.1.2) using the oligo package (v3.6) from the Bioconductor library [25]. Gene and ProbeSet annotation was performed using moex10sttranscriptcluster.db (v3.6) and moex10stprobeset.db (v3.6) packages. Data were normalized using the RMA algorithm [26]. Data were assessed for distributional homogeneity and visualized using the BPG package [27] with the lattice (v0.20–33) and latticeExtra (v0.60–26) packages in R. Raw data is available in GEO at GSE126328, and in the TCDD transcriptomics package (v2.2.5) for R (download from: https://labs.oicr.on.ca/boutros-lab/tcdd-transcriptomics)[28].

### Alternative splicing and statistical analysis

Only ProbeSets with annotation core databases were used for the analysis. Differential abundance analysis was performed using the limma package (v3.2) for R. Linear modeling was performed to contrast each TCDD dose to control. Standard errors of coefficients were adjusted using an empirical Bayes model. Model-based *t*-tests were applied to test for significance, followed by false discovery rate adjustments for multiple testing. To detect alternative splicing events, an entropy base measure for splicing prediction was performed using the ARH package as well as the exon variations within a gene [29].

### Real-time quantitative PCR (qPCR) analysis

Complementary DNA (cDNA) for the qPCR assay was prepared from 3 μg of total liver RNA using random primers and SuperScript First-Strand Synthesis (Invitrogen, Carlsbad, CA, USA). The real-time polymerase reaction (PCR) was performed in a StepOne Real-Time PCR System with TaqMan Universal PCR Master Mix (Applied Biosystems, Branchburg, NJ, USA) according to the manufacturer’s protocol. Relative transcript abundance was quantified using the comparative threshold cycle (Ct) method. The probes used for each exon were obtained from Integrated DNA Technologies (IDT, Skokie, IL, USA). The gene encoding the *18S* ribosomal RNA (rRNA, endogenous) with identification number ID Mm00507222_s1 (IDT) was used to normalize the results.

### Statistical analysis

Real Time quantitative PCR results are presented as the mean values ± standard deviation (S.D.) of three replicates. The statistical significance of the data was evaluated using the Student’s t-test. In all cases, the differences between groups were considered to be statistically significant when the *p* value was less than 0.05.

## Results

Alterations in the splicing process can result in significant deleterious effects on human health. Therefore, identifying agents with the potential to alter alternative splicing is necessary. TCDD are known to impact the transcriptome, however the effects on alternative splicing are unclear. To address this, exon arrays were used to evaluate the alternative splicing of livers from adult male mice exposed to TCDD. The experimental outline is presented in Fig 1.

**Fig 2:**
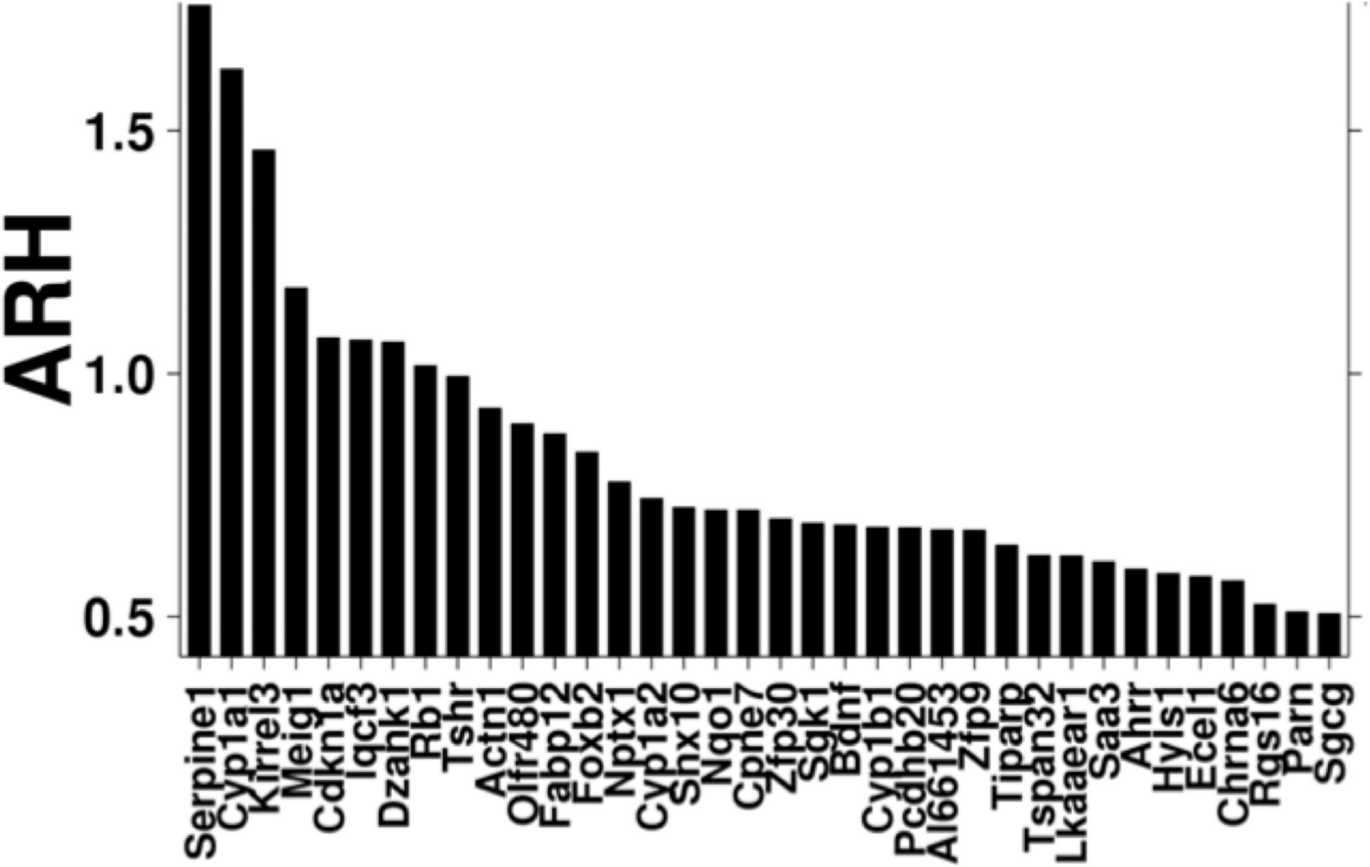
Alternative splicing observed following TCDD exposure. ARH analysis was performed to identify differentially spliced transcripts between 500 μg/Kg TCDD-treated mice and controls. Thirty six genes with ARH values of 0.5 or higher were identified.

After pre-processing the data, splicing assessment was performed *via* ARH analysis. Using a threshold of ≥ 0.5, we identified 36 genes with differential splicing (Fig 2).

**Fig 3:**
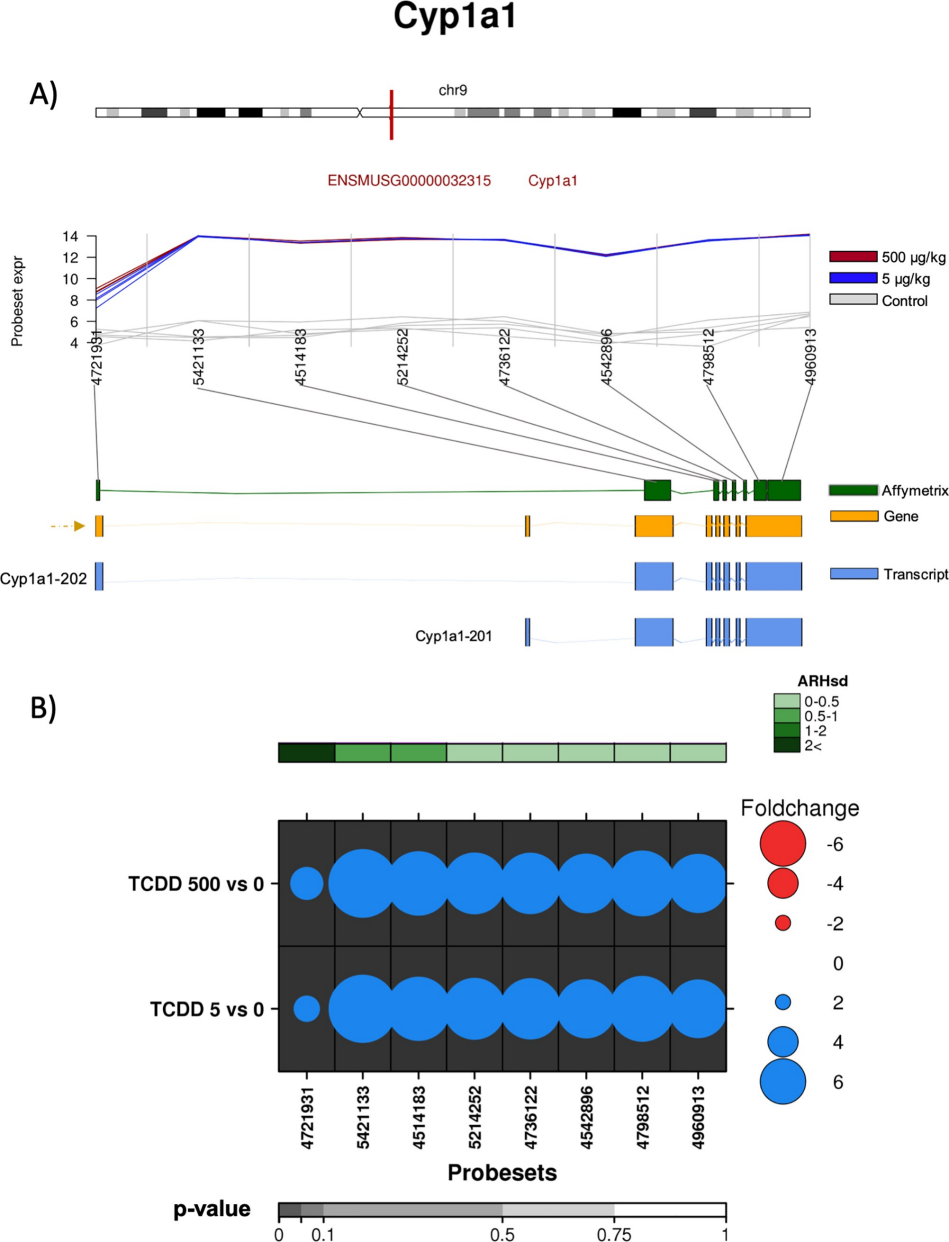
Analysis of the effect of TCDD on exonic abundances of *Cyp1a1*. A) Localization and normalized abundances of ProbeSets targeting distinct exons within the *Cyp1a1* gene. Upper panel: chromosome 9 structure and *Cyp1a1* localization (red line). Middle panel: Log_2_ of the intensities for each of the probes targeting regions within this gene, control (gray lines), low dose TCDD 5 μg/kg (fuchsia lines) and high dose TCDD 500 μg/kg (brown lines). Bottom panel: Comparative structure of known *Cyp1a1* isoforms. Gene reference, including direction of the transcription (yellow), transcripts as reported by Ensembl (blue) and Affymetrix microarray ProbeSets (green). B) RNA abundance of each ProbeSet was contrasted between TCDD treated mouse liver (5 or 500 μg/kg separately) and controls. Dotmap shows the differential abundances for each ProbeSet for each group; such that red dots show reduced abundance following TCDD exposure, while blue shows increased abundance. Dot size represents the magnitude of change. Background shading indicates significant of change, as quantified by the bottom gray bar (FDR adjusted p-value). Top green covariate bar represents the ARH value (with darker shading indicating higher intensity).

From this list, several genes whose abundances were known to be regulated by AhR were identified, including cytochrome P450s 1a1 (*Cyp1a1*), 1a2 (*Cyp1a2*), 1b1 (*Cyp1b1*), NAD(P)H quinone dehydrogenase (*Nqo1*), TCDD inducible poly(ADP-ribose) polymerase (*Tiparp*), aryl hydrocarbon receptor repressor (*Ahrr*) and serum amyloid A (*Saa*) [30, 31]. From these gene sets we determined whether TCDD modified the alternative splicing of the pre-mRNAs of *Cyp1a1* and *Ahrr*. The *Cyp1a1* gene is known to be highly induced following exposure to TCDD. It is composed of 8 exons, 6 of which are coding exons (NCBI) and has been reported to have two different transcripts, Cyp1a1-201 with 7 exons (6 coding exons) and NM_009992.4 (also named Cyp1a1-202) also with 7 exons (6 coding exons) (Fig 3A).

**Fig 4:**
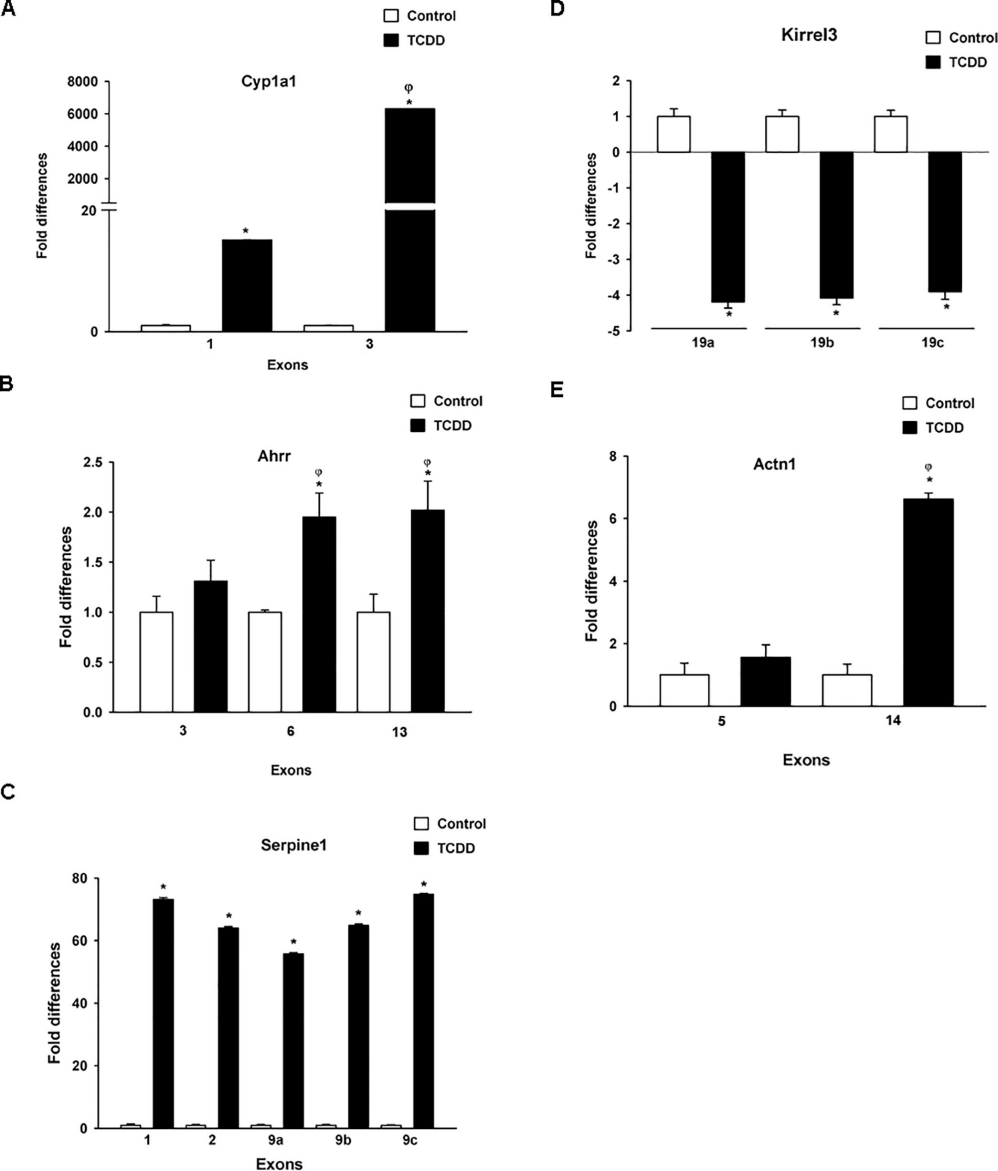
Validation of TCDD effects on exon abundances. Total mRNA was extracted from the liver from mice treated with corn oil or TCDD (500 μg/kg). A) *Cyp1a1* exon 1 (ProbeSet 4721931) and exon 3 (ProbeSet 5421133). B) *Ahrr* exon 3 (ProbeSet 4469533), exon 6 (ProbeSet 5258969) and exon 13 (ProbeSet 4848271). C) *Serpine1* exon 1 (ProbeSet 4403932), exon 2 (ProbeSet 5024287), exon 9a (ProbeSet 4354131), exon 9b (ProbeSet 5079297) and exon 9c (ProbeSet 5082204). D) *Kirrel3* exon 19a (ProbeSet 5043467), exon 19b (ProbeSet 5047088), and exon 19c (ProbeSet 4806289). E) *Actn1* exon 5 (ProbeSet 5008356) and exon 14 (ProbeSet 5597164). mRNA levels were determined by RT-qPCR and normalized to 18S ribosomal RNA. The results are expressed as the mean ± S.E. of samples from three different mice. *p < 0.05, treatment *vs*. control. ^**ȹ**^*p* < 0.05, between treatments.

According to our analysis, at both high and low dose of TCDD (5 and 500 μg/kg), we detected consistently increased abundance of all *Cyp1a1* exons. Interestingly exon 1 showed considerably lower abundance than other exons (Fig 3B), indicating that TCDD treatment changed the relative abundance of the splice variant predominantly to variant 2 (NM_001136059.2). This variant excluded exon 1, which alters the 5’ UTR. These results may also suggest an alternative transcriptional start site located between exon 1 and exon 2; however, *in silico* analysis failed to identify XREs in this region (ALGGEN PROMO database [http://alggen.lsi.upc.es/cgi-bin/promo_v3/promo/promoinit.cgi?dirDB=TF_8.3], TH = 85). Validation of the exon abundance was done *via* qRT-PCR using probes for exons 1 and 3. As Fig 4A shows, 500 μg/kg TCDD treatment resulted in ~6,000-fold induction of exon 3. In contrast, less than 20-fold induction was observed for exon 1, confirming the exon array results.

**Fig 5:**
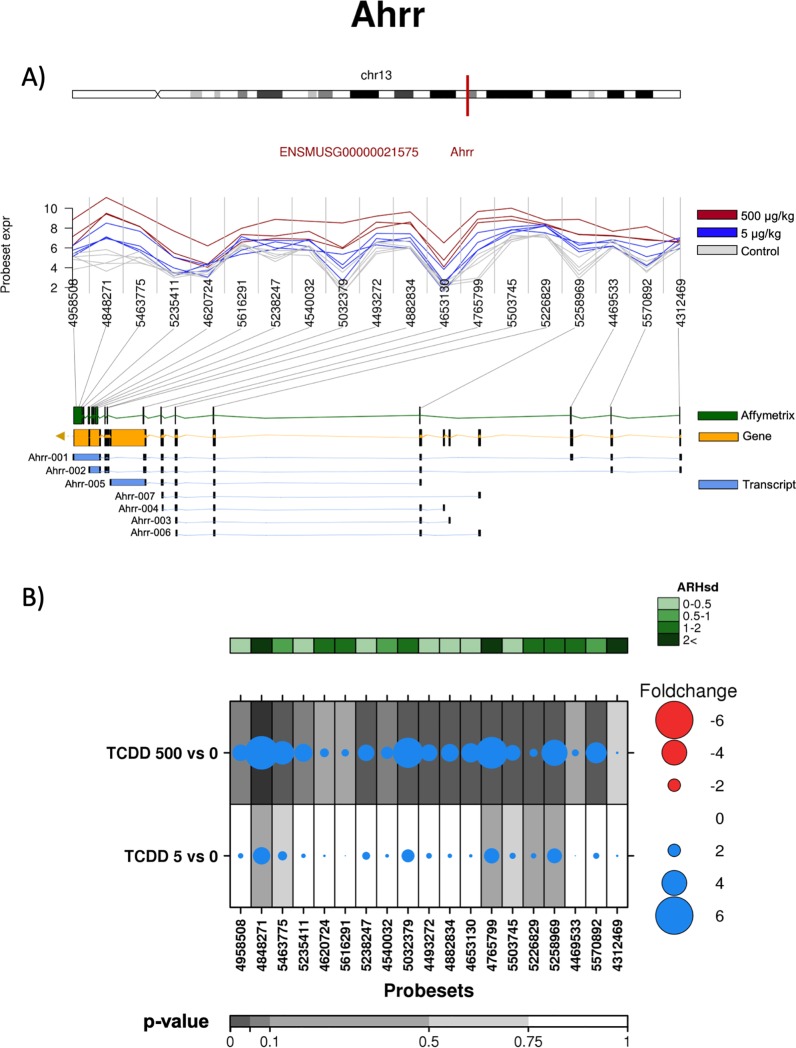
Analysis of the effect of TCDD on exonic abundances of *Ahrr*. A) Localization and normalized abundances of ProbeSets targeting distinct exons within the *Ahrr* gene. Upper panel: chromosome 13 structure and *Ahrr* localization (red line). Middle panel: Log_2_ of the intensities for each of the probes targeting regions within this gene, control (gray lines), low dose TCDD 5 μg/kg (fuchsia lines) and high dose TCDD 500 μg/kg (brown lines). Bottom panel: Comparative structure of known *Ahrr* isoforms. Gene reference, including direction of the transcription (yellow), transcripts as reported by Ensembl (blue) and Affymetrix microarray ProbeSets (green). B) RNA abundance of each ProbeSet was contrasted between TCDD treated mouse liver (5 or 500 μg/kg separately) and controls. Dotmap shows the differential abundances for each ProbeSet for each group; such that red dots show reduced abundance following TCDD exposure, while blue shows increased abundance. Dot size represents the magnitude of change. Background shading indicates significant of change, as quantified by the bottom gray bar (FDR adjusted p-value). Top green covariate bar represents the ARH value (with darker shading indicating higher intensity).

Another interesting gene identified as differentially abundant by ARH was *Ahrr*. The *Ahrr* gene is encoded by 13 exons (Fig 5A), and Ensembl, identified 7 different splice variants for this gene. Based on differential abundance of ProbeSets, we observed preferential expression following TCDD treatment of a splice variant that excluded exons 1 and 3, but included exon 13 (Fig 5B).

This transcript does not correspond to any reported splice variant, so may represent a novel isoform, or a complex combination of isoforms. The RT-qPCR verifies that after TCDD treatment the abundance of exon 3 is indeed lower than that of exons 6 and 13 (Fig 4B).

**Fig 6:**
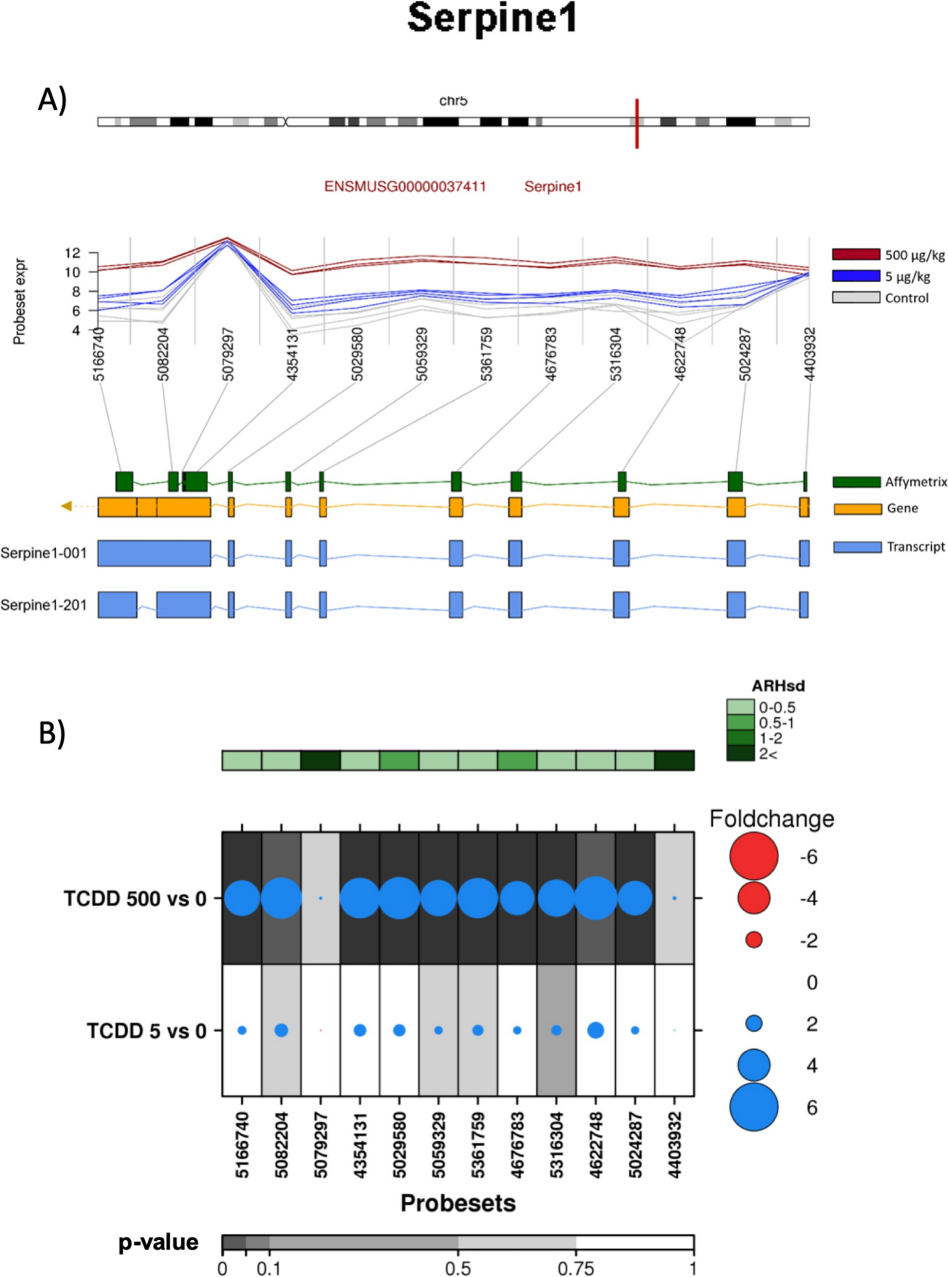
Analysis of the effect of TCDD on exonic abundances of *Serpine 1*. A) Localization and normalized abundances of ProbeSets targeting distinct exons within the *Serpine 1* gene. Upper panel: chromosome 5 structure and *Serpine 1* localization (red line). Middle panel: Log_2_ of the intensities for each of the probes targeting regions within this gene, control (gray lines), low dose TCDD 5 μg/kg (fuchsia lines) and high dose TCDD 500 μg/kg (brown lines). Bottom panel: Comparative structure of known *Serpine 1* isoforms. Gene reference, including direction of the transcription (yellow), transcripts as reported by Ensembl (blue) and Affymetrix microarray ProbeSets (green). B) RNA abundance of each ProbeSet was contrasted between TCDD treated mouse liver (5 or 500 μg/kg separately) and controls. Dotmap shows the differential abundances for each ProbeSet for each group; such that red dots show reduced abundance following TCDD exposure, while blue shows increased abundance. Dot size represents the magnitude of change. Background shading indicates significant of change, as quantified by the bottom gray bar (FDR adjusted p-value). Top green covariate bar represents the ARH value (with darker shading indicating higher intensity).

In addition to known AHR-core genes, ARH detected significant differential splicing of 29 other genes. Of these, *Serpine1* and *Kirrel3* demonstrated the highest ARH index. The *Serpine1* gene encodes for plasminogen activator inhibitor 1. It contains 9 exons, and two transcripts for this gene have been reported, Serpine1-001 and Serpine1-201 (Fig 6A).

The splice variant corresponds to a cassette-exon 9 skipping. Exon array analysis indicates that TCDD treatment increased abundance of all ProbeSets, excluding the 4403932 ProbeSet located at exon 1 and the 5079297 ProbeSet at exon 9 (Fig 6B). Results from qRT-PCR validation demonstrated that TCDD increased the abundance of all exons tested, including exons 1, 2 and 9. The latter was determined by the use of three different ProbeSets. With respect to the magnitude of their induction, no significant differences were observed between the exons analyzed (Fig 4C).

**Fig 7:**
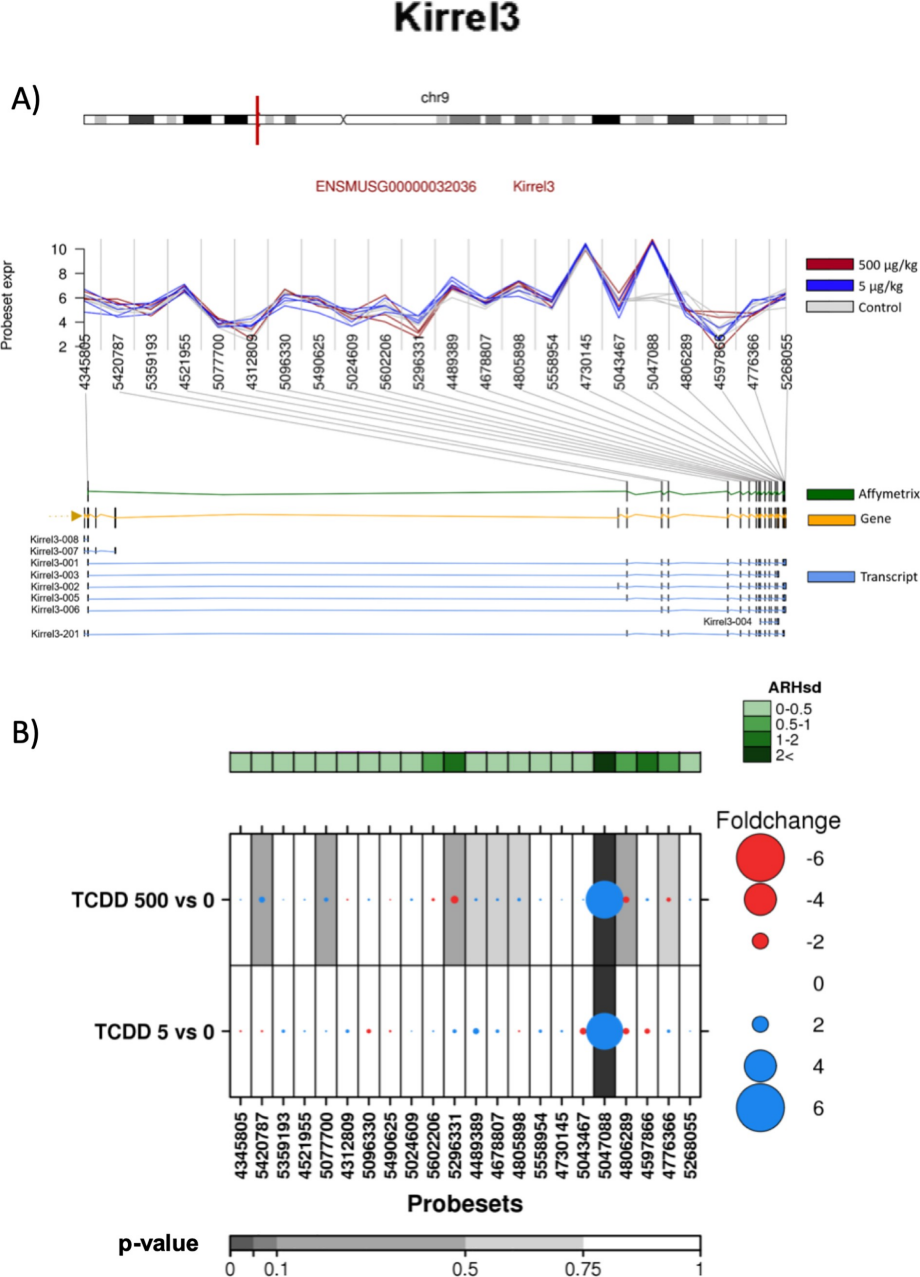
Analysis of the effect of TCDD on exonic abundances of *Kirrel3*. A) Localization and normalized abundances of ProbeSets targeting distinct exons within the *Kirrel3* gene. Upper panel: chromosome 9 structure and *Kirrel3* localization (red line). Middle panel: Log_2_ of the intensities for each of the probes targeting regions within this gene, control (gray lines), low dose TCDD 5 μg/kg (fuchsia lines) and high dose TCDD 500 μg/kg (brown lines). Bottom panel: Comparative structure of known *Kirrel3* isoforms. Gene reference, including direction of the transcription (yellow), transcripts as reported by Ensembl (blue) and Affymetrix microarray ProbeSets (green). B) RNA abundance of each ProbeSet was contrasted between TCDD treated mouse liver (5 or 500 μg/kg separately) and controls. Dotmap shows the differential abundances for each ProbeSet for each group; such that red dots show reduced abundance following TCDD exposure, while blue shows increased abundance. Dot size represents the magnitude of change. Background shading indicates significant of change, as quantified by the bottom gray bar (FDR adjusted p-value). Top green covariate bar represents the ARH value (with darker shading indicating higher intensity).

*Kirrel3* represents the most complex gene on which we focused. The protein encoded by the *Kirrel3* gene is Kirrel-like nephrin family adhesion molecule 3. It contains 19 exons, and 9 different splice variants have been identified (Fig 7A).

Exon array data indicated that TCDD treatment only induced the abundance of the 5047088 ProbeSet located at exon 19. In contrast, ProbeSets 4806289 and 5043467, located in the same exon adjacent to 5047088 ProbeSet, did not exhibit any change after TCDD treatment (Fig 7B), suggesting that dioxin treatment induced the alternative splicing of exon 19. However, the RT-qPCR analysis indicated that TCDD treatment resulted in a decrease in the abundance of all three regions located on exon 19, without differences between them (Fig 4D).

**Fig 8:**
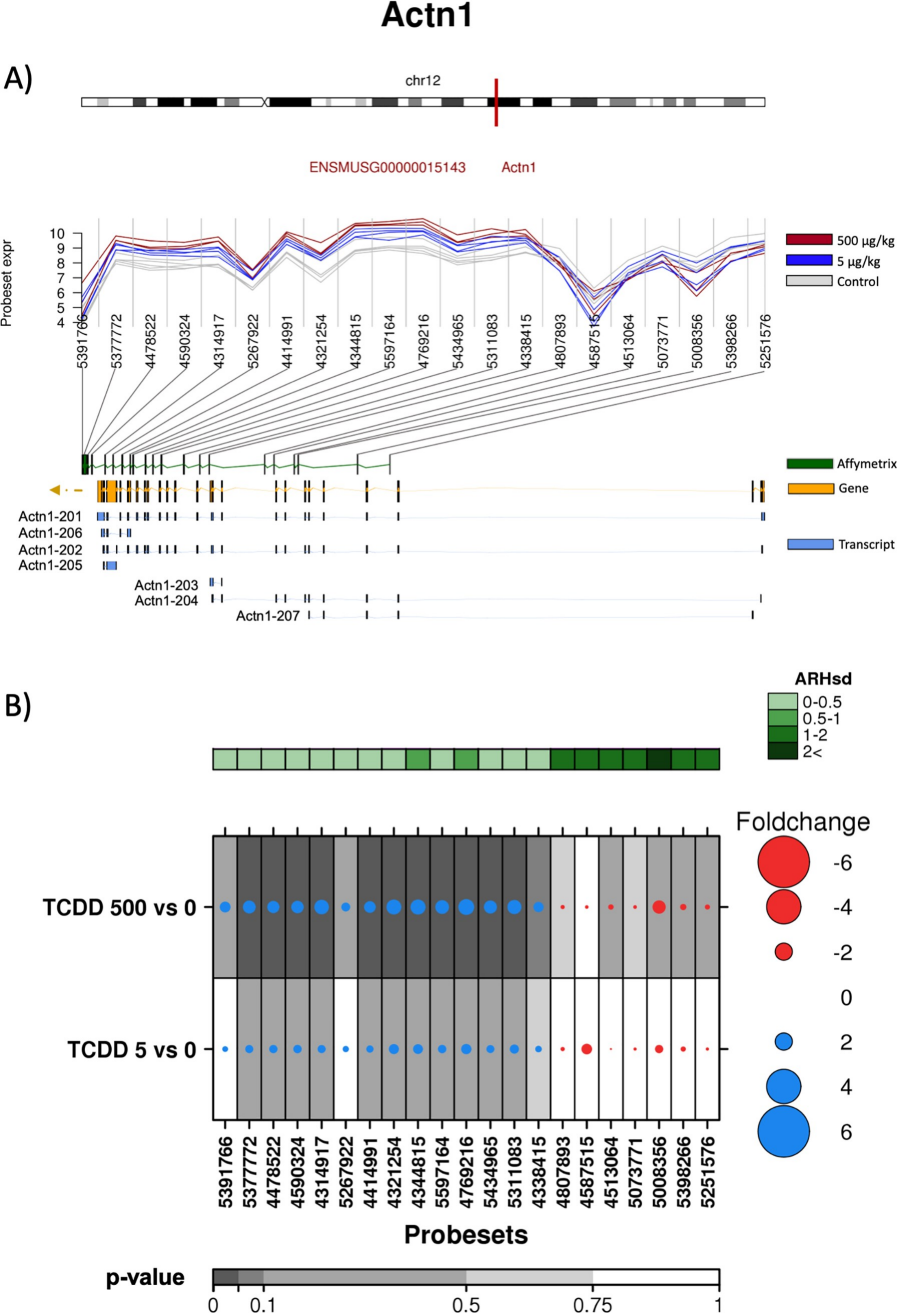
Analysis of the effect of TCDD on exonic abundances of *Actn1*. A) Localization and normalized abundances of ProbeSets targeting distinct exons within the *Actn1* gene. Upper panel: chromosome 12 structure and *Actn1* localization (red line). Middle panel: Log_2_ of the intensities for each of the probes targeting regions within this gene, control (gray lines), low dose TCDD 5 μg/kg (fuchsia lines) and high dose TCDD 500 μg/kg (brown lines). Bottom panel: Comparative structure of known *Actn1* isoforms. Gene reference, including direction of the transcription (yellow), transcripts as reported by Ensembl (blue) and Affymetrix microarray ProbeSets (green). B) RNA abundance of each ProbeSet was contrasted between TCDD treated mouse liver (5 or 500 μg/kg separately) and controls. Dotmap shows the differential abundances for each ProbeSet for each group; such that red dots show reduced abundance following TCDD exposure, while blue shows increased abundance. Dot size represents the magnitude of change. Background shading indicates significant of change, as quantified by the bottom gray bar (FDR adjusted p-value). Top green covariate bar represents the ARH value (with darker shading indicating higher intensity).

Finally, the effect of TCDD on *α-Actn1* alternative splicing was evaluated. *α-Actn1* gene encodes for alpha actinin 1, and contains 22 exons and has 7 identified splicing variants (Fig 8A).

The exon array data suggested that TCDD induced the abundance of a *α-Actn1* variant constituted predominantly by the region encompassing exons 11 to 20 (Fig 8B). These results were supported by RT-qPCR, which indicated a 6-fold induction of exon 14 upon TCDD exposure, while no effect was observed on exon 5 (Fig 4E).

## Discussion

Exposure to TCDD results in a wide range of deleterious effects on human health by altering cell processes, such as the cell cycle and differentiation [32], among many others. To date, it has not been shown whether toxicants like TCDD can alter splice site selection and lead to changes in transcript variant abundances. In the current study, the effect of TCDD on alternative splicing was evaluated through the use of exon arrays. ARH analysis of the exon arrays indicated that the alternative splicing of several liver mouse transcripts was modified by dioxin exposure (Fig 2). Several genes that are transcriptionally regulated by the AhR in response to TCDD exposure were determined to have altered splicing. Two of these (*Cyp1a1* and *Ahrr*), were selected for validation using RT-qPCR. In humans, several CYP1A1 splice variants have been identified, some with functional effects. A variant with deletion of exon 6 has been identified in the human brain, but not in the liver, and it is unable to bioactivate PAHs [33]. In contrast, a CYP1A1 splice variant formed via excision of an 84-bp intron within exon 2 is enzymatically active and is localized exclusively in the nucleus and mitochondria [34]. In mice, a variant that affects the functionality and/or localization of the protein has not yet been reported. The present data indicates that TCDD promotes the abundance of transcript Cyp1a1-201, but it also induces the abundance of the 5’ UTR variant (Cyp1a1-202). Both transcript variants generate the same protein; therefore CYP1A1 enzyme function should remain the same.

Regarding *Ahrr*, it has been reported a human splice variant resulting in modifications of AHRR functions. This variant, not yet included in NCBI database, has been identified as the active form of this protein and lacks exon 8. It is present several tissues, including human liver, lung, heart, spleen, kidney and brain [35]. No report has indicated that any of mouse splice variants result in modifications of *Ahrr* functions. Based on the exon array analysis and the RT-qPCR data indicates that after TCDD treated samples have lower abundance of exon 3 than that of exons 6 and 13, suggesting a novel *Ahrr* splice variant. The functional consequences of this variant require future investigation.

According to the exon array analysis, TCDD treatment also modifies *α-Actn1* alternative splicing, favoring production of a variant lacking the first 8 exons. This observation was partially confirmed by RT-qPCR, which showed that TCDD induces exon 14 abundance 6-fold compared to untreated samples, while no effect was observed for exon 5.

α-Actinin1 is coded by α-*Actn1* gene. Through its ability to interact with actin filaments, α-Actinin1 plays an important role in the regulation of intracellular infrastructure, and therefore in cell adhesion, cytokinesis and cell migration [36]. Calcium binding to the EF-hand domain reduces its affinity for actin filaments. Splicing at the EF-hand domain results in 2 splice variants. Inclusion of exon 19a, but not 19b leads to expression of the non-muscle α-Actinin1 with an active EF-hand domain. In contrast, when exon 19b, but not 19a is included, the calcium-insensitive smooth muscle isoform is expressed [37], which has been associated with cancer cells [38] [39]. As indicated above, TCDD promotes the expression of an α-Actinin1 variant without exon 5, which is located at the *N*-terminal actin binding-domain region [40]. Recently, it has been reported that mutations in the actin-binding domain of α-Actinin1 increase its association with actin [41]. Therefore, the TCDD-induced splice variant might have altered α-Actinin1-actin interactions.

ARH analysis also predicted that TCDD treatment increased the abundance of exon 19 splice variants of *Kirrel3*. However, validation was not consistent with this result. Moreover, RT-qPCR assays indicated that TCDD treatment resulted in a decrease in exon 19 abundance relative to control samples. Although no splicing event was detected, clearly TCDD treatment altered *Kirrel3* abundance. Kirrel3, also known as Neph2, is localized in the pre- and post-synapse in the hippocampus. In particular, Kirrel3 regulates the output specificity of hippocampal DG axons [42], and disruption of its locus has been associated with neurodevelopmental disorders [43]. *Kirrel3* null mice present a reduction in mossy fibers filipodia formation in the hippocampus, resulting in the hyperexcitability of CA3 neurons [44]. Furthermore, a previous study showed that TCDD decreased the side of the pyramidal mossy fiber field of the hippocampus [45]. Therefore, our current results suggest that the effect of TCDD on the mossy fiber field of the hippocampus is through decreasing *Kirrel3* abundance.

Validation of *Serpine1* by qRT-PCR did not show a splicing event as the ARH analysis predicted. However, TCDD treatment induced the expression of exons 1, 2 and 9 more than 50-fold when compared to nontreated samples. The *Serpine1* gene encodes the plasminogen activator inhibitor type I (PAI-1), which inhibits the activation of plasminogen into plasmin, and therefore the proteolysis of proteins in connective tissue, basement membranes and blood clots. The present data suggest that TCDD, through the induction of *Serpine1*, might alter mechanisms involved in tissue remodeling, contributing to the development of several pathologic processes, such as metastasis and intravascular thrombosis [46] [47].

Finally, although the ARH method correctly predicted the alternative splicing of some pre-mRNAs, the analysis also resulted in false positive data as with *Kirrel3* and *Serpine1*, pointing out the need for the improvement of the prediction methods used to evaluate alternative splicing when exon arrays are used. RNA sequencing is also used for the identification of alternative splicing. Both methods have advantages and disadvantages. Nazrev and collaborators in a comparative analysis conclude that combining the use of both platforms for the identification of alternative splicing will be more reliable [48].

In conclusion, this study demonstrated that TCDD exposure leads to modify the alternative splicing of several transcripts. The mechanism of these effects is still unknown and will require future investigation focused on the effects of TCDD on spliceosome assembly, on the recognition of alternative exons, and/or on the levels of splicing factors. Additionally, more studies will be necessary to elucidate whether the AhR plays a role in these splicing changes.
